# Prioritizing strategies for comprehensive liver cancer control in Asia: a conjoint analysis

**DOI:** 10.1186/1472-6963-12-376

**Published:** 2012-10-30

**Authors:** John FP Bridges, Liming Dong, Gisselle Gallego, Barri M Blauvelt, Susan M Joy, Timothy M Pawlik

**Affiliations:** 1 Department of Health Policy and Management, Johns Hopkins Bloomberg School of Public Health, 624 N. Broadway, Room 689, Baltimore, MD, 21205, USA; 2 Institute for Global Health, School of Public Health and Health Sciences, University of Massachusetts, Amherst, MA, 102 Hasbrouck University of Massachusetts, Amherst, MA, 01003, USA; 3 Department of Surgery, Johns Hopkins Hospital, 600 North Wolfe Street, Harvey 611, Baltimore, MD, 21287, USA

**Keywords:** Hepatocellular carcinoma (HCC), Liver cancer, Strategies, Public policy, Conjoint analysis, Prioritization, Discrete choice experiment

## Abstract

**Background:**

Liver cancer is a complex and burdensome disease, with Asia accounting for 75% of known cases. Comprehensive cancer control requires the use of multiple strategies, but various stakeholders may have different views as to which strategies should have the highest priority. This study identified priorities across multiple strategies for comprehensive liver cancer control (CLCC) from the perspective of liver cancer clinical, policy, and advocacy stakeholders in China, Japan, South Korea and Taiwan. Concordance of priorities was assessed across the region and across respondent roles.

**Methods:**

Priorities for CLCC were examined as part of a cross-sectional survey of liver cancer experts. Respondents completed several conjoint-analysis choice tasks to prioritize 11 strategies. In each task, respondents judged which of two competing CLCC plans, consisting of mutually exclusive and exhaustive subsets of the strategies, would have the greatest impact. The dependent variable was the chosen plan, which was then regressed on the strategies of different plans. The restricted least squares (RLS) method was utilized to compare aggregate and stratified models, and t-tests and Wald tests were used to test for significance and concordance, respectively.

**Results:**

Eighty respondents (69.6%) were eligible and completed the survey. Their primary interests were hepatitis (26%), hepatocellular carcinoma (HCC) (58%), metastatic liver cancer (10%) and transplantation (6%). The most preferred strategies were *monitoring at-risk populations* (p<0.001), *clinician education* (p<0.001), and *national guidelines* (p<0.001). Most priorities were concordant across sites except for three strategies: *transplantation infrastructure* (p=0.009) was valued lower in China, *measuring social burden* (p=0.037) was valued higher in Taiwan, and *national guidelines* (p=0.025) was valued higher in China. Priorities did not differ across stakeholder groups (p=0.438).

**Conclusions:**

Priorities for CLCC in Asia include *monitoring at-risk populations*, *clinician education*, *national guidelines, multidisciplinary management*, *public awareness and centers of excellence*. As most priorities are relatively concordant across the region, multilateral approaches to addressing comprehensive liver cancer would be beneficial. However, where priorities are discordant among sites, such as transplantation infrastructure, strategies should be tailored to local needs.

## Background

Hepatocellular carcinoma (HCC), the major histological subtype of primary liver cancer, is the third leading cause of cancer death worldwide [1-3]. In 2008, an estimated 749,000 people were diagnosed with liver cancer and 695,000 people died from it worldwide. The Asia-Pacific region accounted for approximately 75% of known new cases and deaths [3,4]. Hence, controlling liver cancer in Asia is especially important for reducing its global burden.

Most Asian governments have implemented policies and laws to prevent Hepatitis B Virus (HBV) and Hepatitis C Virus (HCV) infection, seeking to control HCC by targeting important risk factors. Universal hepatitis B vaccination, the most effective method to prevent Hepatitis B [5], has been implemented in the majority of Asian countries [6]. Top-down management of medical settings, especially blood centers and blood banks, also ensures the safety of blood products and reduces blood-transmitted HBV and HCV infection [7]. Meanwhile, screening programs for HBV infection, HCV infection and HCC have also been implemented in various ways. Routine HBsAg and anti-HCV tests are offered to voluntary blood donors in all Asian countries, and antenatal exams are offered in most countries [6]. Periodic hepatitis testing among high risk populations is conducted through national programs in Japan, South Korea and Taiwan [6,8-10]. Public education campaigns in most countries also play a major role in increasing the public’s and physicians’ awareness of hepatitis and liver cancer [6,11].

Although the above strategies help to reduce the spread of hepatitis to a large extent, HCC incidence and mortality rates are still increasing in Asia, mainly due to the long natural history from hepatitis to HCC, and the large existing population of HBV and HCV carriers [12]. Strategic national health plans specific to HCC control are needed, but there is a paucity of literature devoted to the optimal design of comprehensive liver cancer control (CLCC).

The WHO provides some guidance for the creation of comprehensive cancer control programs that can offer some guidance for the creation of a CLCC [13]. Similar targeted comprehensive cancer control plans have been advocated and programs implemented for breast, cervical, and colorectal cancer cancer [14-16]. To successfully implement CLCC, plans should reflect current needs [17,18] and incorporate the views of a wide array of stakeholders [13].

The objective of this study was to identify priorities among multiple strategies for CLCC. Specifically we engaged stakeholders involved in clinical, policy, and advocacy activities related to CLCC in China, Japan, South Korea and Taiwan to document priorities and to test if they were concordant across the region and the stakeholder groups. These results are of interest to health care professionals and regulators in Asia, as well as their counterparts in other countries as they can contribute to national or regional efforts to implement CLCC. Our contribution is also important to a wider audience as it demonstrates a transparent and theoretically grounded approach to involving stakeholders in priority setting [19].

### Priority setting methods

If resources were unlimited, international and national decision-makers could immediately adopt all available CLCC strategies that could be expected to have an impact based on existing evidence [20-26]. Implementation is limited by a range of political, financial, human resource and time constraints, such that possible strategies have to be prioritized even if it is envisioned that all strategies will eventually be adopted [27,28].

Cost-effectiveness analysis (CEA) provides one way to set priorities that focuses on allocating resources to where they are (potentially) most beneficial [29]. CEA has been criticized for inadequately incorporating the view of stakeholders [30], ignoring many costs and benefits that fall outside the health sector [31], and inaccurately assessing the benefits of programs [32]. Furthermore, as a decision-making tool, it requires a great deal of information about the costs and effects of programs which may not be available and often lacks transparency [33].

Other processes exist to study priorities that are less restrictive than CEA and, more importantly, allow for a more transparent participation of stakeholders in decision making. Traditionally deliberative process approaches have been used that rely upon qualitative methods [34] and Delphi approaches [35,36]. While such approaches have an important role, they are grounded in normative political theory, rather than some explicit theory of prioritization [37]. Furthermore, the results of such analyses can be subject to the biases of the researchers or facilitators [38], by dominant respondents [39] or misinterpretation [40]. While modern approaches to deliberation, such as citizen juries [41] or Analytic Hierarchy Process (AHP) [42] have overcome some of these concerns, they are also more focused on decision making rather than the scientific study of priorities that may inform decision makers.

While simple rating and ranking approaches can be applied to priority setting exercises, they are subject to a range of biases and complexities [43]. The self-explicated methods, which combine ratings and rankings in a multiplicative way in an attempt to minimize the biases of rating and ranking alone, is one method that has potential for priority setting, especially in the presence of a large number of factors [17,18,44].

Stated-preference methods, such as conjoint analysis [45,46] and best-worst scaling (BWS) [47], offer a potentially superior alternative to simple rating and ranking methods as they ensure people have to make tradeoffs [43]. Furthermore, as these methods can utilize random utility theory (RUT), they are more consistent with economic [48] and psychological [49] theories of choice.

In conjoint analysis, the factors that are to be assessed (which we might call objects to differentiate them from attributes that may vary across defined levels) are presented in competing subsets (traditionally two) and respondents are asked to make some judgment or choice as to which of the sets are better [50]. The benefit of this approach is that it reinforces the notion that multiple objects are likely to incorporated in the eventual policy. The downfall of this approach is that experimental designs used to create such subsets are often based on orthogonal or D-efficient designs that will lead to unbalanced numbers of objects in each subset [50]. Furthermore, such designs are generally “main-effects” which implies that key interactions between the objects are not estimated. Hence, one could not determine if objects were considered complements or substitutes in the minds of respondents.

In BWS case 1, which is known as the object case [51], respondents are presented with subsets of the objects and asked which subset is the best (with regards to some criteria such as “which will have the greatest effect?”) and which is worst on those same criteria. The primary benefit of this approach is that it is a relatively easy decision that is relatable to real life situations. Researchers often use a balanced incomplete block design (BIBD) which ensures that there are the same number of objects in each task (at the cost of orthogonality) [51,52]. The limitations of this method are that it enforces the notion that one of the objects is the best (rather than promoting that a portfolio of objects may be optimal) and that, like conjoint analysis, interactions are rarely explored.

## Methods

Stakeholders are often consulted during policy processes because they can provide critical insight overlooked by more objective evaluation methods [53]. The WHO recommended that policy makers incorporate stakeholders’ input as part of a systematic and transparent evaluation of priorities [19]. For the purpose of this study we have chosen conjoint analysis as a means of involving stakeholders in the prioritization of strategies that could be incorporated into a CLCC plan. While this approach has limitations, as detailed above, we thought it important to reinforce the notion that a CLCC plan needed to incorporate multiple strategies.

In a previously published pilot study on this topic, the conjoint analysis approach was shown to be both feasible and functional even in a very low sample size (n=20) [54]. Several limitations of the original study were identified and were corrected in this present application of the technique. First, only a single stakeholder group (clinicians) was included; here we also have engaged both policy makers and advocates. Second, while a sample size of 20 was sufficient to measure aggregate priorities, we have aimed to examine heterogeneity across sites in Asia or across different stakeholder subgroups. Third, based on the pilot results, and comparison with qualitative data, several modifications to the survey instrument were recommended in the previous publication [54] and all have been incorporated here. Finally, the previous publication used publication guidelines designed for qualitative research, and not specifically targeted for conjoint analysis. While other guidelines for conjoint analysis have been proposed [55-58], this paper has adhered to recently published guidelines for the application of conjoint analysis in health care presented by the International Society for Pharmacoeconomics and Outcomes Research (ISPOR) [59].

### Research question, perspective and rationale

The primary research question was to identify stakeholder priorities across multiple strategies for CLCC in Asia, to assess if these differed across clinical, policy, and advocacy stakeholders and to explore the concordance of priorities across China, Japan, South Korea and Taiwan. Respondents were asked to use their own perspective in judging which strategies were better for their country. As discussed above, conjoint analysis was chosen as it is a valid method for assessing priorities and preference heterogeneity, and as it reinforced the notion that multiple strategies were needed if a CLCC were to be successful. No priors were used for the purpose of experimental design, and the hypotheses were centered on identifying which factors were deemed valuable and whether differences in priorities could be identified across stakeholders or countries.

### Selection of CLCC strategies

Data from previous qualitative interviews (n=20) with international liver cancer clinicians were analyzed using Interpretive Phenomenological Analysis to identify these strategies [54]. A pilot was then conducted with clinicians from China, Japan, and Korea to assess the importance of these strategies in comparison with the qualitative data [54]. Based on this analysis, the wording of the strategies surfaced as a potential limitation of the pilot study. In order to avoid any ambiguity in interpretation in the present study, the strategy “Measuring the social burden of liver cancer” was relabeled “Measuring incidence, prevalence and burden of liver cancer” and the strategy “Improved risk-assessment and referral by primary care” was relabeled “Early risk assessment in primary care”. An explanation of the eleven strategies presented in this conjoint analysis is presented in Table 1.

**Table 1:** Attributes included in the study

**Label**	**Strategy**	**Description**
Access to treatments	Improved access to recommended treatments	All liver cancer patients and people that are at-risk of liver cancer have access to recommended medical care, including doctors’ ability to request screening and tests for liver function and asymptomatic disease through liver cancer prevention and treatment, paid by governmental and/or private health insurance.
Centers of excellence	Centers of excellence for liver cancer	Referral of patients with liver cancer and other liver diseases that increase risk of liver cancer, to medical centers or research institutions with major liver disease departments that treat liver cancer and/or conduct the latest research in liver cancer. Specialized liver cancer centers to provide coordinated surveillance, treatment and research within a national liver cancer program.
Clinician Education	Education of primary care physicians and hepatologists about HCC	Education of related healthcare providers (primary care physicians, internists, gastroenterologists, hepatologists) about liver cancer, such as the importance of early risk assessment, management of hepatic diseases to prevent progression to liver cancer.
Measuring social burden	Measuring incidence, prevalence and burden of liver cancer	Measuring the health care costs or burden to the society, compared to screening, earlier detection and management of liver cancer patients or at-risk people.
Monitoring at-risk population	Continuous surveillance of at-risk populations	Monitoring at-risk populations to detect liver diseases and liver cancer at an early stage, including the stratification of disease risk and hepatic disease surveillance in those at high risk of HCC.
Multidisciplinary management	Multidisciplinary management of HCC	Due to the complex nature of liver cancer and multiple contributing factors, a highly skilled multidisciplinary team approach is necessary for managing liver cancer surgically and non-surgically, as well as managing patients with hepatitis and other diseases that significantly increase the risk of developing liver cancer.
National guidelines	National standards and guidelines	The establishment and maintenance of nationwide, evidence-based standards and guidelines for the adoption of prevention, treatment and surveillance of liver cancer.
Public awareness	Organized disease advocacy and public awareness	Widespread public awareness of risk factors and prevention for liver disease as well as liver cancer in the general population, including organized consumer liver disease and liver cancer advocacy.
Research infrastructure	Increased infrastructure for translational research	Increased infrastructure, including capacity and qualified personnel, to conduct translational, clinical and basic research in all stages of liver cancer, from prevention to end-stage disease treatment and care.
Risk Assessment and referral	Early risk assessment in primary care	Earlier and improved risk assessment and screening by primary healthcare providers (general practitioners, internists, gastroenterologists) and immediate referral of patients with diagnosed liver disease and/or liver cancer to medical experts specializing in hepatic diseases and /or HCC.
Transplantation infrastructure	Transplantation infrastructure and allocation	Improved transplantation infrastructure and allocation of livers, including total liver transplants as well as partial transplants from living donors; sufficient highly skilled surgical and other healthcare providers, latest transplant techniques and technology.

### Construction of choice tasks

**Figure 1:**
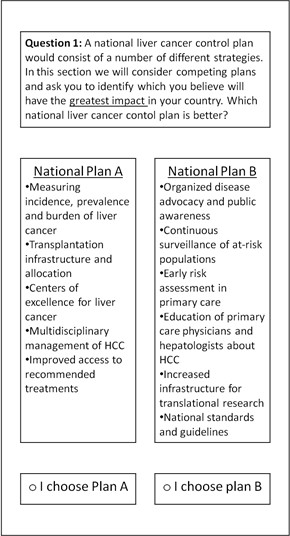
**An example of a conjoint analysis choice task** .

These eleven strategies, which were considered as being present or absent in a potential plan, were assigned to one of two plans within each of the conjoint analysis choice tasks by creating two mutually exclusive and exhaustive subsets. This ensured all eleven strategies appear at least once in each choice task, but not twice. While there is some emerging evidence that triplets have some benefits over paired choice tasks [60], the use of pairs is currently the standard for applications in health [61]. As no country has adopted CLCC we did not incorporate a status quo into the choice tasks. An example of a choice task is illustrated in Figure 1.

### Experimental design

A main-effects orthogonal experimental design was used to create the choice tasks [62]. This ensured zero collinearity among strategies and that importance of each strategy could be estimated independently. This 2^11 design involved eleven columns of binary numbers, one for each strategy, and twelve rows, which were translated into the twelve conjoint-analysis choice tasks presented in the survey. Here a strategy was placed on the left in a task if the orthogonal array indicated a zero and on the right if the orthogonal array indicated a one. Alternative strategies were also considered, including designs based on D-optimality and D-efficiency [59], but these approaches would have led to identical designs under the same assumptions. The properties of the experimental design were assessed, confirming that the design was orthogonal (both in terms of profiles and tasks), 100% D-Optimal for main effects in a paired experiment, and that level balance and zero overlap were achieved. Finally, with a design leading to only 12 choice tasks, the respondent burden was deemed acceptable based on experience with the pilot study and given current standards [59,61].

### Preferences elicitation and survey design

In each of the 12 choice tasks, stakeholders were asked to choose the CLCC plan that they believed would have a more significant impact in their country. As such, a forced-choice preference elicitation procedure was used that is consistent with the underlying theory [48,49]. Respondents were not asked to justify or explain their preferred strategy, and for simplicity strength of preference or confidence in their answer was not explored.

Prior to preference elicitation, respondents were provided sufficient motivation about the strategies to facilitate an appropriate response. An example choice task was also presented to confirm all respondents understood how to complete the choice tasks correctly.

The questionnaire also collected background information including respondents’ areas of involvement and level of involvement in liver cancer control. The properties of the questionnaire were examined during the pilot study and relevant modifications were noted above.

### Sampling strategy, ethical considerations and data collection

Based on the experience from our pilot, a sample of 20 respondents per country was used or n=80 overall. Although this is relatively small for a conjoint analysis [61], we had to acknowledge that relevant experts in liver cancer are not in endless supply. A strategy of purposive quota sampling was used to ensure that a relative balance of stakeholder types was recruited.

Clinical, policy, and advocacy stakeholders were purposively selected equally from China, Japan, South Korea and Taiwan. Potential respondents were actively involved in liver cancer prevention and control activities as identified by authoritative literature, medical and public health networks, peer referral and leadership roles in government agencies, national centers or medical institutes/associations. Study inclusion and exclusion criteria are shown in Table 2. Exclusion criteria ensured all potential respondents were familiar with national liver cancer control policy and practice.

**Table 2:** Inclusion and exclusion criteria

**Role**	**Inclusion criteria**	**Exclusion criteria**
Clinical	Oncologists, surgeons, radiologists, other HCC and hepatobiliary specialists, hepatologists, pathologists, and other specialists who may be involved in HCC prevention, diagnosis, treatment and care, or leaders of major medical institutions (including cancer and other liver disease centers).	Not board certified, certified for less than one year, practicing medicine for less than 3 years, living/practicing in country for less than 3 years.
Policy	Individuals in government, NGOs or other agencies involved in public education, awareness and prevention related to liver disease and liver cancer; national formularies and reimbursement decision-making; the development of policy and/or guidelines for the control of liver cancer; or those involved in policy related to liver transplantation.	Less than 1 year’s experience in liver cancer and related fields; those not directly involved in policies impacting liver cancer prevention and control; or those with primary responsibilities as (and who otherwise primarily identify themselves as liver cancer) clinicians, advocates or in non-policy related roles.
Advocacy	Recognized by liver cancer patients, physicians or policy leaders for their national advocacy role; significant consumer/patient advocacy of liver disease; leadership role in a nationally recognized advocacy group; evidence of an active media and/or publication history that is targeted to reach liver cancer patient or at-risk consumer populations.	Those with primary responsibilities as clinicians or in non-advocacy related roles; those whose scope of advocacy is limited to local environs, i.e. with little/no national impact or recognition by peers, clinical and policy leaders.

All respondents were informed about the study and its potential risks and benefits. Respondents participated voluntarily and were not reimbursed for participation. The study was deemed exempt from the Institutional Review Board (IRB) review at Johns Hopkins Bloomberg School of Public Health.

#### Data collection

Potential respondents were contacted for participation via email or mail. Follow-up phone calls were made to those who did not respond within two weeks. If there was still no response after a maximum of four reminders, they were considered as “no response”. Eligible respondents were invited to complete the survey by email or telephone. The survey was administered as an interviewer-assisted face-to-face interview in English and in the respondents’ native languages where necessary between October 2010 and April 2011.

### Statistical analysis, inference and validity

Our data were structured so that an observation was obtained for each of the 12 tasks across the 80 respondents, leading to 960 observations (e.g. 12x80). The primary outcome (dependent variable) in the analysis was the liver cancer control plan selected by the participant for each task. Here each choice was coded as 1 if the respondent chose the plan on the left or 0 if they choose the plan on the right. The independent variables were a set of eleven dichotomous variables indicating which tasks were present. A strategy was coded as 1 if it was presented in the plan on the left and −1 if it was presented in the plan on the right. The coding strategy is required (as opposed to a traditional dichotomous variable) because the absence of the strategy on the left meant that it was automatically on the right. This can be seen in a linear representation of the underlying theory [48].

The difference in the value placed on the options (say *A* and *B*) can be expressed as simply subtraction between the value placed on the attributes presented in *A* and *B* respectively (denoted *X*^*A*^ and *X*^*B*^), as presented in eq. 1.

(1) $$\Delta V=V\left(X^{A}\right)-V\left(X^{B}\right)$$

Given that we have assumed linearity and have eleven independent variables, we can think of the differences between the values as being defined as:

(2) $$\Delta V=\sum_{i=1}^{11}\beta_{i}X_{i}^{A}-\sum_{i-1}^{11}\beta_{i}X_{i}^{B}$$

As *ΔV* is unobserved, we need a mechanism to approximate it. As seen in eq. 3, our dichotomous variable indicating which card was shown can approximate this difference and be regressed against difference in the cards using ordinary least squares. The restricted regression model is thus:

(3) $$Y=\alpha +\sum_{i-1}^{11}\beta_{i}\left(X_{i}^{A}-X_{i}^{B}\right)$$

Regressing the respondents’ choices on the strategies produced a set of coefficients for the strategies which will lead to the estimation of the *β*_*i*_, which will assess the marginal valuation of each of the eleven strategies. These estimated coefficients therefore showed the relative importance of different strategies in a hypothetical liver cancer control plan. The higher the value of the coefficients, the higher the strategy was prioritized. Coefficients with positive signs indicated the strategies were valued, while negative signs indicated respondents were opposed to those strategies.

To account for variation across our sample, say by country, we can stratify the model by allowing the *β* to vary by strategy *i* and country *j*, leading to:

(4) $$Y=\alpha +\sum_{j=1}^{4}\sum_{i-1}^{11}\beta_{\mathrm{ij}}\left(X_{i}^{A}-X_{i}^{B}\right)+\varepsilon$$

Here we chose not to consider differences in the constant term across the strata, as there was not theoretical reason to suggest that the strata were particularly biased to choose one side of the experiment over the other.

From eq. 4, differences across countries for each strategy are tested by applying restricted least squares (RLS) [63], using the *β*_*i*1_ = *β*_*i*2_ = *β*_*i*3_ = *β*_*i*4_ restriction, where the RLS will produce a Wald statistic that tests the significance of this restriction, and the overall test of differences across countries is conducted by testing all restrictions on the eleven strategies at once. The same model specification and Wald tests were used for the model stratified by stakeholder type.

Using the RLS based on ordinary least squares (OLS) estimates has a number of advantages over alternative strategies. First, OLS does not require the assumption of independence of irrelevant alternatives [48]. Second, the difference between logit and OLS is generally found to be small in conjoint analysis experiments [55,64]. Third, given our main-effects orthogonal design and zero priors on parameters, a linear model can be considered more appropriate [64-66].

Robust standard errors were estimated to account for the clustering of multiple observations from each respondent. Analyses were conducted using Stata 11.2 for Windows (StataCorp LP, College Station, TX). The t-test and Wald test were used to test for significance and concordance respectively. Pearson Chi-Square tests were used to test for differences in stakeholder characteristics across the region. All the parameter estimates are multiplied by 100 to aid interpretation.

## Results

**Figure 2:**
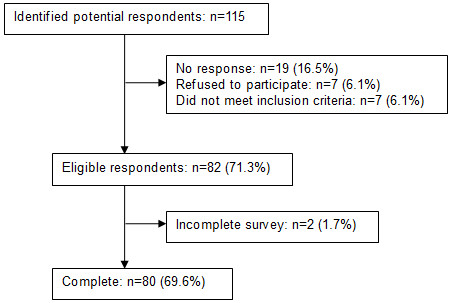
**Study recruitment** .

Eighty respondents were eligible and completed the survey (69.6% completion rate). As shown in Figure 2, 115 potential participants were invited to participate in the study. Of these, 19 (16.5%) did not respond, 7 (6.1%) refused to participate, 7 (6.1%) did not meet the inclusion criteria and 2 (1.7%) did not complete the survey.

Table 3 shows stakeholders’ characteristics. Their primary roles were clinical (60%), policy (25%) and advocacy (15%). Their main areas of involvement were hepatitis (26.3%), hepatocellular carcinoma (57.5%), metastatic liver cancer (10.0%), and transplantation (6.3%). Respondents were involved in liver cancer control at international (15.0%), local/municipality (6.3%), national (56.3%) and regional/provincial (22.5%) levels.

**Table 3:** **Characteristics of respondents** (**n**=**80**) **stratified by site**

	**All**	**China**	**Japan**	**South Korea**	**Taiwan**	**p**-**value**
**Primary Role**, %						
***Clinicians***	60.0	15.0	15.0	15.0	15.0	0.196
Hepatologist	18.8	2.5	5.0	7.5	3.8	
Oncologist	21.3	10.0	2.5	1.3	7.5	
Radiologist	3.8	1.3	1.3	1.3	0.0	
Surgeon	12.5	0.0	8.3	4.2	0.0	
Other	3.8	0.4	1.1	1.1	1.1	
***Policy makers***	25.0	6.3	6.3	6.3	6.3	0.167
Governmental	18.8	5.0	6.3	5.0	2.5	
Non-Governmental	6.3	1.3	0.0	1.3	3.8	
***Advocates***	15.0	3.8	3.8	3.8	3.8	0.518
Disease advocacy	5.0	0.0	1.3	1.3	2.5	
Media/Spokesperson	3.8	1.3	1.3	0.0	1.3	
Patient advocacy	6.3	2.5	1.3	2.5	0.0	
**Area of Involvement**, %						0.002**
Hepatitis	26.3	5.0	11.3	3.8	6.3	
HCC	57.5	17.5	13.7	16.3	10.0	
Metastatic liver cancer	10.0	2.5	0.0	0.0	7.5	
Transplantation	6.3	0.0	0.0	5.0	1.3	
**Level of Involvement**, %						0.029*
International	15.0	3.8	3.8	5.0	2.5	
Local/municipality	6.3	5.0	0.0	1.3	0.0	
National	56.3	13.7	11.3	17.5	13.7	
Regional/provincial	22.5	2.5	10.0	1.3	8.8	

*p<0.05, **p<0.01.

In the aggregate analysis, all eleven strategies were positively valued and statistically significant (at p<0.01) except for *transplantation infrastructure*. The most preferred strategy was *monitoring at-risk populations* (coefficient=13.75). The next preferred strategy was *clinician education* (11.04), followed by *national guidelines* (8.96), *multidisciplinary management* (8.33), *public awareness* (8.13), *centers of excellence* (6.86), *risk assessment* (5.42), *access to treatments* (5.21), *measuring social burden* (5.21), and research infrastructure (3.96). The coefficient for *transplantation infrastructure* (0.42) was not significantly different from zero (p=0.848), indicating it was not expected to have a significant impact. The constant term was not significantly different from 0.5 (50.2; p=0.866), indicating no bias towards plans on the left or the right.

In the subset analysis, we stratified the priorities by sites and stakeholder groups, as shown in Table 4 and Table 5. Priorities differed across countries (p<0.001). In China, *national guidelines* (14.17; p<0.001) was valued highest and *transplantation infrastructure* was valued lowest (−8.33; p=0.025), as shown in Table 4. In Japan and South Korea, *monitoring at-risk populations* (16.67 and 17.08 respectively; both p<0.001) was identified as the top priority, but their least preferred strategies diverged: in Japan, *risk assessment* was valued lowest (0.83; p=0.732) while *transplantation infrastructure* was the lowest in South Korea (−0.42; p=0.877). In Taiwan, *measuring social burden* (12.92; p<0.001) was valued highest and *research infrastructure* was valued lowest (1.25; p=0.677).

**Table 4:** Priorities stratified by site

**Variable**	**Aggregate**	**China**	**Japan**	**South Korea**	**Taiwan**	**Chi-squaredp**-**value**
	***Coef****(****SE****)*	***Coef****(****SE****)*	***Coef****(****SE****)*	***Coef****(****SE****)*	***Coef****(****SE****)*	
Monitoring at-risk populations	13.75***	9.17**	16.67***	17.08***	12.08***	0.20
	(1.5)	(3.1)	(2.7)	(3.3)	(2.5)	
Risk Assessment	5.42***	5.83*	0.83	7.08*	7.92**	0.18
	(1.3)	(2.4)	(2.4)	(2.9)	(2.4)	
Access to treatments	5.21***	10.83***	5.83*	1.25	2.92	0.11
	(1.4)	(3.0)	(2.4)	(2.7)	(3.2)	
National guidelines	8.96***	14.17***	10.00***	7.92**	3.75	0.02*
	(1.4)	(2.8)	(3.3)	(2.7)	(2.0)	
Research infrastructure	3.96**	4.17	6.67*	3.75	1.25	0.63
	(1.4)	(2.5)	(2.8)	(2.5)	(3)	
Centers of excellence	6.86***	5.83*	10.00***	6.25*	5.42*	0.60
	(1.5)	(3.9)	(2.5)	(1.9)	(3.1)	
Multidisciplinary management	8.33***	7.50**	11.67***	6.25*	7.92**	0.44
	(1.4)	(2.4)	(2.1)	(3.4)	(2.7)	
Clinician education	11.04***	7.50**	10.83***	15.42***	10.42***	0.15
	(1.3)	(2.3)	(3.2)	(2.5)	(2.6)	
Public awareness	8.13***	3.33	6.67*	11.25***	11.25***	0.11
	(1.4)	(2.7)	(2.8)	(2.7)	(2.6)	
Transplantation infrastructure	0.42	−8.33	5.83	−0.42	4.58	0.01**
	(1.5)	(3.6)	(2.4)	(2.7)	(2.7)	
Measuring Social Burden	5.21***	2.50	2.50	2.92	12.92***	0.04*
	(1.4)	(2.5)	(2.7)	(2.4)	(3.1)	

*p<0.05, **p<0.01, ***p<0.001, aggregate model R^*2*^*=0.269, p<0.001, stratified model R*^*2*^*=.320.*

**Table 5:** Priorities stratified by stakeholder role

**Variable**	**Aggregate**	**Clinicians**	**Policy Makers**	**Advocates**	**Chi-squaredp**-**value**
	***Coef(SE)***	***Coef(SE)***	***Coef(SE)***	***Coef(SE)***	
Monitoring at-risk populations	13.75***	14.76***	14.58***	8.33*	0.28
	(1.5)	(2.0)	(2.6)	(3.6)	
Risk Assessment	5.42***	5.73**	2.92	8.33*	0.47
	(1.3)	(1.6)	(2.7)	(3.6)	
Access to treatments	5.21***	4.34*	6.25*	6.94	0.76
	(1.4)	(1.7)	(3.6)	(3.6)	
National guidelines	8.96***	9.55***	7.08*	9.72**	0.79
	(1.4)	(1.7)	(3.3)	(3.6)	
Research infrastructure	3.96**	5.03**	−1.25	8.33*	0.08
	(1.4)	(1.6)	(2.9)	(3.3)	
Centers of excellence	6.86***	7.81***	4.58	6.94	0.66
	(1.5)	(1.9)	(3.0)	(3.9)	
Multidisciplinary management	8.33***	9.20***	7.92**	5.56	0.52
	(1.4)	(2.0)	(2.3)	(2.5)	
Clinician education	11.04***	11.28***	8.75**	13.89***	0.35
	(1.3)	(1.9)	(2.4)	(2.5)	
Public awareness	8.13***	9.20***	3.75	11.11**	0.15
	(1.4)	(1.8)	(2.5)	(3.8)	
Transplantation infrastructure	0.42	1.56	−2.92	1.39	0.47
	(1.5)	(2.1)	(3.1)	(3.6)	
Measuring social burden	5.21***	3.3	8.75**	6.94	0.25
	(1.4)	(1.6)	(3.0)	(4.8)	

*p<0.05, **p<0.01, ***p<0.001, model R^*2*^*=.287*.

Table 5 compares the results among stakeholder groups. Priorities differed across stakeholder groups (p=0.001). *Monitoring at-risk populations* was most preferred by clinical and policy stakeholders (14.76 and 14.58 respectively; both p<0.001), while advocates valued *clinician education* (13.89; p<0.001) highest. When it came to least preferred strategy, all stakeholder groups agreed on *transplantation infrastructure* (1.56, -2.92, and 1.39 for clinical, policy and advocacy stakeholders respectively; all p>0.05).

As there were slight differences among groups, we tested for concordance across sites and stakeholder groups. Most priorities were concordant across sites except for three strategies: *transplantation infrastructure* (p=0.009) was valued lower in China than Japan (−8.33 vs 5.83; p=0.002) and Taiwan (−8.33 vs 4.58; p=0.006); *measuring social burden* (p=0.037) was valued significantly higher in Taiwan than in China, Japan and Taiwan (12.92 vs 2.5, 2.5, and 2.92 respectively; all p<0.05); and *national guidelines* (p=0.025) with higher valuations in China than Taiwan (14.17 vs 3.75; p=0.003). In contrast, priorities were concordant across stakeholder groups with no differences for any of the strategies (all p>0.05).

## Discussion

Strategies viewed as having the highest priority in Asia were *monitoring at-risk populations*, *clinician education*, *national guidelines*, *multidisciplinary management*, *public awareness, and centers of excellence.* Priorities were relatively concordant among the three groups of stakeholders (clinical, policy, and advocacy) and across the region. This said, three strategies, *transplantation infrastructure, measuring social burden*, and *national guidelines* received different priorities across the region. The fact that most priorities are shared across the four study sites provides justification for utilizing these strategies in the sites we studied, and suggests the findings could be generalized to other Asian countries with similar situations.

The strategy of *monitoring at-risk populations* is ranked as the top priority, consistent with some existing actions. Three of the four sites already have monitoring systems in place, suggesting either that the existing system is valued and should continue in a new comprehensive liver cancer control plan, or that the existing system could be improved. South Korea, Japan and Taiwan started national cancer screening programs in the 1990s with liver cancer screening as an important component [8,10,67]. Especially in Japan, where HCV infection is a major etiological cause of liver cancer, a HCV screening program has been implemented since the late 1980s [10]. Japan’s National Project against Hepatitis and HCC specifically focuses on screening hepatitis carriers to prevent them from developing liver cancer and also to detect early-stage liver cancer patients [10]. Screening is an effective method to control liver cancer and improve prognosis, and might be expected to be one of the most important strategies for liver cancer control given the large existing populations of HBV and HCV carriers in most Asian countries [9].

The finding of heterogeneity in transplantation infrastructure across sites is interesting. Respondents from China valued this strategy extremely low, compared to those from the other three sites. This phenomenon is consistent with evidence of barriers to liver transplantation in China. Although demand for liver transplantation has been growing rapidly in China, ethical criteria and governing legislation have not yet been fully established, which deters the utilization of liver transplantation in liver cancer management [32,33]. Without legislation, the quality and safety of liver transplantation are not supervised and guaranteed for patients who undergo surgery, and the legal rights of healthcare providers are not protected [68,69]. Faults in the regulatory system also leave space for illegal organ trades [68]. In addition, liver cancer transplantation is an expensive surgical procedure that is not covered by health care insurance in China, so affordability is another barrier to full utilization [69]. Thus, it is reasonable that stakeholders from China did not consider it a high priority despite the fact that it is considered an effective treatment for some liver cancer patients.

Our study demonstrated that conjoint analysis, a stated preference method, can be utilized to prioritize strategies for a comprehensive disease control plan. It combines qualitative research methods with quantitative methods, and provides a comprehensive way to explore stakeholders’ judgments in the policy decision-making process. Compared to CEA, the most prevalent prioritization tool in health economics, conjoint analysis has several advantages [31]. First, it can take into consideration all possible outcomes (including risks and costs) of health policies rather than just using a single measurement to rank them [31]. Conjoint analysis can integrate preferences from different stakeholders whose views are important when considering policy interventions, hence the results are a useful step towards developing consensus. Indeed, our study found good consensus among stakeholders from clinical, policy, and patient advocacy roles, who together should have a good understanding of decision making throughout their health systems. Second, the theoretical basis of conjoint analysis is less controversial than CEA, making it easier to justify the method to stakeholders such as physicians and policy makers [31].

This study demonstrated the usefulness of the conjoint-analysis method in studying stakeholders' priorities for CLCC strategies, although there are a number of limitations that need to be considered. First, given that the data come from subjective responses, there might be some variations in preference due to respondents’ specific positions, geography, or experience that do not truly reflect societal preferences and cannot be assessed with such a small sample. We tested concordance across the three selected stakeholder groups, and the results showed there was no statistically significant heterogeneity. However, to ensure representativeness and validity, additional important stakeholders should be included in future studies, such as patients and health policy researchers, and larger samples should be considered in countries such as China where there may be large differences in disease burden and resources within the country. In addition, if the sample size permitted, it would be useful to incorporate a latent class analysis in order to identify sources of heterogeneity that may not be captured by the characteristics that we chose to investigate.

A second limitation is that our study was conducted in countries where English is not the native language. Although the questionnaire was provided in both English and respondents’ first languages, there might be some misunderstandings due to translation.

The third limitation is that the generalizability of the study is limited by the study sites that were selected. Among our four sites, Japan, South Korea and Taiwan are high-income countries, while China is different in terms of health care resources, health financing and people’s socio-economic status. As most Asian countries are low or middle income countries, the results may not be applicable to all Asian countries. Further studies could be conducted in low or middle income parts of Asia to examine whether strategies are prioritized differently in such areas.

A fourth limitation of using conjoint analysis to assess priorities is that respondents may have simply preferred plans with more strategies than others. While all strategies were positively correlated with the number of strategies, this was uniform across the strategies, so bias in the prioritization would be difficult. This said, we did re-estimate the aggregate model by holding constant the number of attributes (which also required dropping the intercept to avoid the dummy variable trap). We did identify that there was a significant effect associated with the number of strategies presented in the model (p<0.001). In correcting for this bias, we found that only five strategies were significantly different to zero and positive (*monitoring at-risk populations*: 9.2, p<0.001; *clinician education*: 6.5, p<0.001; *national guidelines*: 4.4, p=0.003; *multidisciplinary management*: 3.8, p=0.008; and *public awareness*: 3.6, p=0.011) and one was statistically significant and negative (*transplantation infrastructure*: -4.1, p=0.011). In comparing these results to those reported above, the exact prioritization across the strategies was estimated. Future research is needed on separating the number of objects in a conjoint from the marginal effects estimated for each parameter.

Finally, the study sample size remains low and may lack generalizability to other Asian countries. The robustness of the results obtained in the aggregate model and the stratified models confirm the findings of the pilot study that small sample sizes can be used [54]. This said, the lack of statistical difference between the groups can be attributed to the low sample size. While simulation techniques may provide some benefit in overcoming low sample sizes, such methods would also have to correct for the underlying differences in scale that may be present across the strata.

## Conclusions

This study has used a systematic and transparent method to produce a prioritization of effective strategies for liver cancer control in Asia. The finding of concordance across four sites, despite differences in liver cancer etiology and resources across sites, suggests that the priorities are applicable to other parts of the region. The results of this study constitute a ready-to-use, prioritized plan for liver cancer control based on the views of key stakeholders. The next step is for decision makers to implement the priority strategies as comprehensive national plans and to instigate cross-national or regional collaborations that can use the similarities in priorities to improve liver cancer control across the region.

## Abbreviations

HCC: Hepatocellular carcinoma; HBV: Hepatitis Bvirus; HCV: Hepatitis Cvirus; WHO: World Health Organization; CEA: Cost effectiveness analysis; RLS: restricted least squares; OLS: ordinary least squares; CLCC: comprehensive liver cancer control; BWS: Best worst scaling.

## Competing interests

The authors have no competing interests.

## Authors' contributions

JB and BB conceptualized the study, designed the study instrument, and made substantial contributions to the data interpretation and writing of the paper. LD, GG and SJ participated in data analysis and drafting of the final manuscript. TP provided subject matter advice. All authors read and approved the final manuscript.

## Pre-publication history

The pre-publication history for this paper can be accessed here:

http://www.biomedcentral.com/1472-6963/12/376/prepub

## References

[B1] Srivatanakul P Sriplung H Deerasamee S Epidemiology of liver cancer: an overview Asian Pac J Cancer Prev 2004 5 118 125 15244512

[B2] Yang JD Roberts LR Hepatocellular carcinoma: a global view Nat Rev Gastroenterol Hepatol 2010 7 448 458 10.1038/nrgastro.2010.100 20628345 PMC3926946

[B3] Ferlay J Shin HR Bray F Forman D Mathers C Parkin DM GLOBOCAN 2008 v1.2, Cancer incidence and mortality worldwide: IARC CancerBase No. 10 2010 International Agency for Research on Cancer, Lyon http://globocan.iarc.fr

[B4] Jemal A Bray F Center MM Ferlay J Ward E Froman D Global cancer statistics CA Cancer J Clin 2011 61 69 90 10.3322/caac.20107 21296855

[B5] World Hepatitis Alliance Viral hepatitis: global policy 2010 http://worldhepatitisalliance.org/Policy/2010PolicyReport.aspx

[B6] Mohamed R Desmond P Suh DJ Amarapurkar D Gane E Guangbi Y Hou JL Jafri W Lai CL Lee CH Lee SD Lim SG Guan R Phiet PH Piratvisuth T Sollano J Wu JC Practical difficulties in the management of hepatitis B in the Asia-pacific region J Gastroenterol Hepatol 2004 19 958 969 10.1111/j.1440-1746.2004.03420.x 15304110

[B7] Korean Ministry of Health and Welfare Blood management policy 2012 http://english.mohw.go.kr/front_eng/jc/sjc0102mn.jsp?PAR_MENU_ID=100302&MENU_ID=10030203

[B8] National cancer control programs in Korea J Korean Med Sci 2007 22 S3 S4 18077892 10.3346/jkms.2007.22.S.S3 PMC2694372

[B9] Tsukuma H Tanaka H Ajiki W Oshima A Liver Cancer and its Prevention Asian Pac J Cancer Prev 2005 6 244 250 16235981

[B10] Yoshizawa H National prevention of hepatocellular carcinoma in Japan based on epidemiology of hepatitis C virus infection in the general population Intervirology 2006 49 7 17 10.1159/000087257 16166783

[B11] Jun DW Cho YK Sohn JH Lee CH Kim SH Eun JR A study of the awareness of chronic liver diseases among Korean adults Korean J Hepatol 2011 17 99 105 10.3350/kjhep.2011.17.2.99 21757980 PMC3304642

[B12] Poon D Anderson BO Chen LT Tanaka K Lau WY Van Cutsem E Singh H Chow WC Ooi LL Chow P Khin MW Koo WH Management of hepatocellular carcinoma in Asia: consensus statement from the Asian oncology summit 2009 Lancet Oncol 2009 10 1111 1118 10.1016/S1470-2045(09)70241-4 19880065

[B13] WHO National cancer control programmes: policies and managerial guidelines 2002 2 World Health Organization, Geneva

[B14] Anderson BO Distelhorst SR Guidelines for international breast health and cancer control–implementation Cancer 2008 113 Suppl 8 2215 2216 18837029 10.1002/cncr.23980

[B15] World Health Organization, Department of Reproductive Health and Research and Department of Chronic Diseases and Health Promotion Comprehensive cervical cancer control: a guide to essential practice 2006 World Health Organization, Geneva

[B16] Steger C Daniel K Gurian GL Petherick JT Stockmyer C David AM Miller SE Public policy action and CCC implementation: benefits and hurdles Cancer Causes Control 2010 21 2041 2048 10.1007/s10552-010-9668-5 21086034 PMC3006649

[B17] Bridges J Joy S Gallego G Kudo M Han K-H Ye S-L Cheng A-L Blauvelt B Needs for hepatocellular carcinoma (HCC) control policy in the Asia-pacific region Asian Pac J Cancer Prev 2011 12 2585 2591 22320959

[B18] Bridges J Joy S Gallego G Blauvelt B Geschwind JF Pawlik T Priorities for hepatocellular carcinoma (HCC) control: a comparison of policy needs in five European countries J Comp Pol Anal 2012 14 352 368

[B19] Oxman AD Schünemann HJ Fretheim A Improving the use of research evidence in guideline development: 2. priority setting Health Res Policy Syst 2006 4 14 20 10.1186/1478-4505-4-14 17134481 PMC1702532

[B20] Kudo M Izumi N Kokudo N Matsui O Sakamoto M Nakashima O Kojiro M Makuuchi M Management of hepatocellular carcinoma in Japan: consensus-based clinical practice guidelines proposed by the Japan society of hepatology (JSH) 2010 updated version Dig Dis 2011 29 339 364 10.1159/000327577 21829027

[B21] Sharma P Saini SD Kuhn LB Rubenstein JH Pardi DS Marrero JA Schoenfeld PS Knowledge of hepatocellular carcinoma screening guidelines and clinical practices among gastroenterologists Dig Dis Sci 2011 56 569 577 10.1007/s10620-010-1453-5 20978844 PMC3482004

[B22] Jun DW Cho YK Sohn JH Lee CH Kim SH Eun JR A study of the awareness of chronic liver diseases among Korean adults Korean Journal Hepatol 2011 17 99 105 10.3350/kjhep.2011.17.2.99 PMC3304642 21757980

[B23] Mazzaferro V Llovet JM Miceli R Bhoori S Schiavo M Mariani L Camerini T Roayaie S Schwartz ME Grazi GL Adam R Neuhaus P Salizzoni M Bruix J Forner A De Carlis L Cillo U Burroughs AK Troisi R Rossi M Gerunda GE Lerut J Belghiti J Boin I Gugenheim J Rochling F Van Hoek B Majno P Predicting survival after liver transplantation in patients with hepatocellular carcinoma beyond the Milan criteria: a retrospective, exploratory analysis Lancet Oncol 2009 10 35 43 10.1016/S1470-2045(08)70284-5 19058754

[B24] Masuoka HC Rosen CB Liver transplantation for hepatocellular carcinoma: expanding frontiers and building bridges Clin Liver Dis 2011 15 385 393 10.1016/j.cld.2011.03.005 21689620

[B25] Giacomin A Cazzagon N Sergio A Vanin V Farinati F Hepatitis B virus-related hepatocellular carcinoma: primary, secondary, and tertiary prevention Eur J Cancer Prev 2011 20 381 388 10.1097/CEJ.0b013e328346399b 21540746

[B26] Sherman M Burak K Maroun J Metrakos P Knox JJ Myers RP Guindi M Porter G Kachura JR Rasuli P Gill S Ghali P Chaudhury P Siddiqui J Valenti D Weiss A Wong R Multidisciplinary Canadian consensus recommendations for the management and treatment of hepatocellular carcinoma Curr Oncol 2011 18 228 240 21980250 10.3747/co.v18i5.952 PMC3185900

[B27] Sibbald SL Gibson JL Singer PA Upshur R Martin DK Evaluating priority setting success in healthcare: a pilot study BMC Health Serv Res 2010 10 131 144 10.1186/1472-6963-10-131 20482843 PMC2890637

[B28] Goddard M Hauck K Smith PC Priority setting in health - a political economy perspective Health Econ Policy Law 2006 1 79 90 18634704 10.1017/S1744133105001040

[B29] Jamison DT, Breman JG, Measham AR, Alleyne G, Claeson M, Evans DB, Jha P, Mills A, Musgrove P Disease control priorities in developing countries 2006 World Bank, Washington, DC http://files.dcp2.org/pdf/DCP/DCPFM.pdf 21250296

[B30] Bridges JF What can economics add to health technology assessment? please not just another cost-effectiveness analysis! Expert Rev Pharmacoecon Outcomes Res 2006 6 19 24 10.1586/14737167.6.1.19 20528533

[B31] Bridges JF Stated preference methods in health care evaluation: an emerging methodological paradigm in health economics Appl Health Econ Health Policy 2003 2 213 224 15119540

[B32] Liu L Rettenmaier AJ Saving TR Longevity bias in cost-effectiveness analysis Health Econ 2008 17 523 534 10.1002/hec.1309 17990284

[B33] Drummond M Sculpher M Common methodological flaws in economic evaluations Med Care 2005 43 suppl 7 5 14 16056003 10.1097/01.mlr.0000170001.10393.b7

[B34] Mitton C Patten S Evidence-based priority-setting: what do the decision-makers think? J Health Serv Res Policy 2004 9 146 152 10.1258/1355819041403240 15272972

[B35] Dalkey N Helmer O An experimental application of the DELPHI method to the use of experts Manag Sci 1963 9 458 467 10.1287/mnsc.9.3.458

[B36] Viergever RF Olifson S Ghaffar A Terry RF A checklist for health research priority setting: nine common themes of good practice Health Res Policy Syst 2010 8 36 44 21159163 10.1186/1478-4505-8-36 PMC3018439

[B37] Abelson J Forest P-G Eyles J Smith P Martin M Gauvin F-P Deliberations about deliberative methods: issues in the design and evaluation of public participation processes Soc Sci Med 2003 57 239 251 10.1016/S0277-9536(02)00343-X 12765705

[B38] Fitzpatrick R Boulton M Qualitative methods for assessing health care Qual Health Care 1994 3 107 113 10.1136/qshc.3.2.107 10137583 PMC1055206

[B39] Vogt DS Focus groups in psychological assessment: enhancing content validity by consulting members of the target population Psychol Assess 2004 16 3 231 243 15456379 10.1037/1040-3590.16.3.231

[B40] Onwuegbuzie AJ Leech NL Validity and qualitative research: an oxymoron? Qual Quant 2007 41 233 249 10.1007/s11135-006-9000-3

[B41] Armour A Renn O, Webler T, Wiedemann P The citizens’ jury model of public participation: a critical evaluation Fairness and competence in citizen participation 1995 Kluwer Academic Publishers, Dordrecht 175 185 Technology, risk, and society: an international series in risk analysis, vol 10

[B42] Dolan JG Multi-criteria clinical decision support: a primer on the use of multiple criteria decision making methods to promote evidence-based, patient-centered healthcare Patient 2010 3 229 248 10.2165/11539470-000000000-00000 21394218 PMC3049911

[B43] Ryan M Scott DA Reeves C Bate A van Teijlingen ER Russell EM Napper M Robb CM Eliciting public preferences for healthcare: a systematic review of techniques Health Technol Assess 2001 5 1 186 11262422 10.3310/hta5050

[B44] Bridges JF Slawik L Schmeding A Reimer J Naber D Kuhnigk O A test of concordance between patient and psychiatrist valuations of multiple treatment goals for schizophrenia Health Expect 2011 Epub ahead of print 10.1111/j.1369-7625.2011.00704.x PMC5060657 21668795

[B45] Bridges JF Selck FW Gray GE McIntyre JA Martinson NA Condom avoidance and determinants of demand for male circumcision in Johannesburg, South Africa Health Policy Plan 2011 26 298 306 10.1093/heapol/czq064 20961943

[B46] Bridges JFP Searle SC Selck FW Martinson NA Engaging families in the choice of social marketing strategies for male circumcision services in Johannesburg, south Africa Soc Market Q 2010 16 60 76 10.1080/15245004.2010.500443

[B47] Flynn TN Louviere JJ Peters TJ Coast J Best-worst scaling: what it can do for health care research and how to do it J Health Econ 2007 26 171 189 10.1016/j.jhealeco.2006.04.002 16707175

[B48] McFadden D In Frontiers in Econometrics Conditional logit analysis of qualitative choice behavior 1974 Edited by Zarembka P, New York: Academic Press 105 142

[B49] Thurstone LL A law of comparative judgment Psychol Rev 1927 34 273 286

[B50] Finn A Louviere J Determining the appropriate response to evidence of public concern: the case of food safety Journal of Public Policy & Marketing 1992 11 12 25

[B51] Street D Street AP Combinatorics of experimental design 1987 Clarendon Press, Oxford

[B52] Flynn TN Using conjoint analysis and choice experiments to estimate QALY values: issues to consider PharmacoEconomics 2010 28 711 722 10.2165/11535660-000000000-00000 20568837

[B53] WHO Cancer control: knowledge into action. WHO guide for effective programmes 2006 World Health Organization, Geneva 24741742

[B54] Bridges JF Gallego G Kudo M Okita K Han KH Ye SL Blauvelt BM Identifying and prioritizing strategies for comprehensive liver cancer control in Asia BMC Health Serv Res 2011 11 298 309 10.1186/1472-6963-11-298 22047535 PMC3227633

[B55] Green PE Srinivasan V Conjoint analysis in consumer research: issues and outlook J Consum Res 1978 5 103 123 10.1086/208721

[B56] Lancsar E Louviere J Conducting discrete choice experiments to inform healthcare decision making: a user's guide PharmacoEconomics 2008 26 661 677 10.2165/00019053-200826080-00004 18620460

[B57] Ryan M Farrar S Using conjoint analysis to elicit preferences for health care BMJ 2000 320 1530 1533 10.1136/bmj.320.7248.1530 10834905 PMC1118112

[B58] Viney R Lancsar E Louviere J Discrete choice experiments to measure consumer preferences for health and healthcare Expert Rev Pharmacoecon Outcomes Res 2002 2 319 326 10.1586/14737167.2.4.319 19807438

[B59] Bridges JF Hauber AB Marshall D Lloyd A Prosser LA Regier DA Johnson FR Mauskopf J Conjoint analysis applications in health–a checklist: a report of the ISPOR good research practices for conjoint analysis task force Value Health 2011 14 403 413 10.1016/j.jval.2010.11.013 21669364

[B60] Bridges JFP Buttorff C Groothuis-Oudshoorn K Estimating patients' preferences for medical devices: does the number of profiles in choice experiments matter? NBER Working Paper 2011 17482

[B61] Marshall D Bridges JF Hauber B Cameron R Donnalley L Fyie K Johnson FR Conjoint analysis applications in health - how are studies being designed and reported? An update on current practice in the published literature between 2005 and 2008 Patient 2010 3 249 256 10.2165/11539650-000000000-00000 22273432

[B62] Kuhfeld WS Orthogonal arrays 2012 SAS Knowledge Base Papers TS-723. Raleigh, SAS Institute Inc http://support.sas.com/techsup/technote/ts723.html

[B63] Dykstra RL An algorithm for restricted least squares regression J Am Statist Assoc 1983 78 837 842 10.1080/01621459.1983.10477029

[B64] Hauser JR Rao VR In Advances in Marketing Research Conjoint analysis, related modeling, and applications 2004 Progress and Prospects. Edited by Wind Y, Green PE, New York: Springer 141 168

[B65] Angrist JD Pischke J-S Mostly harmless econometrics: an Empiricist’s companion 2009 Princeton University Press, Princeton

[B66] Wang Q Shi G Chan-Halbrendt C Market potential for fine furniture manufactured from low-grade hardwood: evidence from a conjoint analysis in the northeastern united states Forest Prod J 2004 54 19 25

[B67] Chen DS Hepatocellular carcinoma in Taiwan Hepatol Res 2007 37 S101 S105 10.1111/j.1872-034X.2007.00170.x 17877468

[B68] Huang J Ethical and legislative perspectives on liver transplantation in the People’s republic of china Liver Transpl 2007 13 193 196 10.1002/lt.21081 17256779

[B69] Wu J Zheng SS Liver transplantation in china: problems and their solutions Hepatobiliary Pancreat Dis Int 2004 3 170 174 15138103

